# Exosomes Derived from Tumor Cells Initiate Breast Cancer Cell Metastasis and Chemoresistance through a MALAT1-Dependent Mechanism

**DOI:** 10.1155/2022/5483523

**Published:** 2022-06-30

**Authors:** Shuang Tao, Zhengyang Bai, Yaobang Liu, Yali Gao, Jia Zhou, Yangyang Zhang, Jinping Li

**Affiliations:** ^**1**^ Department of Breast Surgery, Wu Jin Hospital of Jiangsu University, Changzhou 213000, China; ^**2**^ Ningxia Medical University, Yinchuan 750004, China; ^**3**^ Department of Surgical Oncology, General Hospital of Ningxia Medical University, Yinchuan 750004, China

## Abstract

**Background:**

Chemoresistance poses a great hindrance in the treatment of breast cancer (BC). Interestingly, exosome (Exo)-mediated transfer of long noncoding RNAs (lncRNAs) has been reported to regulate chemoresistance in diverse diseases. We herein investigate the potential role of lncRNA metastasis-associated lung adenocarcinoma transcript 1 (MALAT1) transferred by BC cell-derived Exo in chemoresistance of BC cells.

**Methods:**

BC-related lncRNAs were identified. Exosomes were isolated and verified from BC cells. The expression patterns of MALAT1 were then examined in the adriamycin (ADR)-sensitive and resistant cells and the isolated Exo, followed by the analysis of the downstream microRNA (miRNA) of MALAT1. The role and mechanism of MALAT1 transmitted by BC cell-derived Exo in BC cell metastasis and chemoresistance were assessed.

**Results:**

MALAT1 was highly expressed in BC cells and their Exo. In addition, MALAT1 delivered by BC cell-derived Exo augmented the malignant properties and chemoresistance of BC cells. Mechanistically, MALAT1 bound to miR-1-3p and limited the miR-1-3p expression, which sequentially targeted the vasodilator-stimulated phosphoprotein (VASP) protein. Moreover, silencing of VASP inhibited the activation of the RAP1 member of RAS oncogene family (Rap1) signaling pathway, which led to the attenuation of BC cell malignant properties and chemoresistance. *In vivo* assay further validated the tumor-promoting effect of Exo-MALAT1 *via* regulation of the miR-1-3p/VASP/Rap1 axis.

**Conclusion:**

Collectively, MALAT1 loaded by BC cell-derived Exo can accelerate BC cell metastasis and chemoresistance *via* disruption of miR-1-3p-mediated inhibition of the VASP/Rap1 signaling axis.

## 1. Introduction

Breast cancer (BC) is not only the most prevalent cancer in women, but also accounts for the one of the highest numbers of cancer-related deaths in female populations across the world [1]. The occurrence of BC can be attributed to a variety of factors, including excessive alcohol intake, physical inactivity, family history, race, genetic traits, and exogenous hormones [2]. Despite the tremendous gains made in regard to screening, diagnosis, and therapeutic modalities against BC, drug resistance and poor prognosis still persist as major clinical challenges [3]. Accordingly, it is imperative to elucidate the underlying mechanism controlling BC progression and chemoresistance, in order to actively develop novel and effective therapeutic targets against BC.

Exosomes (Exos) (30–200 nm in diameter), representing a form of extracellular vesicles, are known to possess a diverse range of cargoes, such as proteins, lipids, nucleic acids, as well as glycoconjugates [4, 5]. Interestingly, cancer cells often produce more Exo than normal cells, whereas cancer cell-derived Exo also exert various roles in cancer diagnosis and treatment, owing to their aforementioned ability to transfer bioactive cargoes, including long noncoding RNAs (lncRNAs) to recipient cells [6]. One such lncRNA, named MALAT1, is frequently enhanced in tumors and metastases, while its overexpression is positively associated with tumor progression and metastasis in numerous cancers, including BC [7]. Recent studies have further highlighted the upregulation of MALAT1 in BC as a potential predictor for the diagnosis [8]. The knockdown of MALAT1 in trastuzumab-resistant BC HER2^+^ cells was previously shown to limit cell invasion, while simultaneously enhancing the sensitivity of BC cells to trastuzumab [9]. On a separate note, epigenetic changes represent an essential process in the development and progression of numerous cancers, unsurprisingly in breast cancer as well [10]. More specifically, alterations in microRNA (miRNA) play key roles in cancer development and progression [11]. Existing evidence has further shown the binding of miR-1-3p to MALAT1 in prostate cancer cells, such that MALAT1 is capable of reducing the expression of miR-1-3p [12]. The latter is particularly noteworthy as downregulation of miR-1-3p expression was previously documented in BC tissues, whereas its upregulation was associated with anticancer effects in BC [13]. Initial findings from the starBase and TargetScan databases highlighted the presence of binding sites between miR-1-3p and vasodilator-stimulated phosphoprotein (VASP). The VASP protein is further established to overexpressed in BC, such that its enhancement contributes to BC cell malignant properties [14]. Moreover, VASP overexpression can also augment the resistance of BC cells to tamoxifen [15]. Furthermore, Ras-related protein 1 (Rap1), a member of the Ras family, is also highly expressed in human BC, while its knockdown results in the reduction of BC cell migration and further sensitizes cells to apoptosis [16, 17]. Recent investigations have also highlighted that changes in Rap1 expression can serve as a predictor for BC response to chemotherapy [18]. Accordingly, we designed the current study in an effort to unveil the role of MALAT1 delivered by BC cell-derived Exo in BC cell metastasis and chemoresistance and its association with miR-1-3p, VASP, and Rap1.

## 2. Materials and Methods

### 2.1. Ethics Statement

The current study was carried out with the approval of the Animal Ethics Committee of General Hospital of Ningxia Medical University and performed in accordance with the Guide for the Care and Use of Laboratory animals published by the US National Institutes of Health. Extensive efforts were undertaken to minimize both the number and suffering of the included animals.

### 2.2. Bioinformatic Analysis

Firstly, the lncACTdb, starBase, RAID, and DIANA databases were retrieved to predict the downstream miRNAs of MALAT1. Subsequently, BC-related gene expression dataset GSE70905 (comprising of 47 normal samples and 47 tumor samples) was downloaded from the Gene Expression Omnibus (GEO) database. Differential analysis was carried out using the R language “limma” package, with |logFoldChange| > 2 and adj.*p* < 0.05 serving as the threshold to obtain the upregulated genes in BC. The false discovery rate method was adopted for correction of the difference *p*value. The starBase and TargetScan databases were retrieved again to obtain the target genes of miR-1-3p. VASP-related genes were additionally retrieved from the GeneMANIA database, and then subjected to KEGG enrichment analysis using the R “clusterprofiler” package. The starBase database was adopted to collect the binding sites between MALAT1, miR-1-3p, and VASP.

### 2.3. Cell Culture

Human breast epithelial cell line MCF-10A, adriamycin (ADR)-sensitive BC cell line MCF-7 (MCF-7/S), and HEK293T cells were all procured from American Type Culture Collection (Manassas, VA). Subsequently, MCF-7 cells were exposed to ADR of increasing concentrations in order to develop BC cell line MCF-7 resistant to 500 nM ADR (MCF-7/ADR). These cells were cultured in Roswell Park Memorial Institute (RPMI) 1640 medium (Gibco, Grand Island, NY) containing 10% fetal bovine serum (FBS; Gibco), 100 U/mL penicillin, and 100 *μ*g/mL streptomycin in a 5% CO_2_ incubator (BB15, Thermo Fisher Scientific Inc., Waltham, MA) at 37°C. Next, the medium for MCF-7/ADR was supplemented with 1.0 *μ*mol/L of ADR, with the culture medium refreshed every 24 h. Afterwards, the cells were detached with 0.25% trypsin (HyClone Laboratories, Logan, Utah) every 72 h, passaged and collected.

### 2.4. Isolation and Identification of Exo

MCF-10A, MCF-7/S, and MCF-7/ADR were cultured in 10% FBS-containing medium (without Exo, which were removed by means of ultracentrifugation at 100000 × *g* and 4°C overnight). After 48 h, the cell culture supernatant was ultracentrifuged to isolate the Exo. Subsequently, the supernatant was centrifuged at 300 × *g* and 4°C for 5 min, then at 2000 × *g* for 10 min, and one more time at 10000 × *g* for 30 min to remove cells and large debris. Following filtration (0.22-*μ*m filter), the supernatant was centrifuged at 140000 × *g* and 4°C for 3 h and then rinsed with 10 mL phosphate-buffered saline (PBS). Next, the pellet was ultracentrifuged at 140000 × *g* and 4°C for 2 h and resuspended in PBS, with the Exo obtained, namely MCF-10A-Exo, MCF-7/S-Exo, and MCF-7/ADR-Exo.

The observation of morphology of the isolated Exo was carried out using a JEM-2000EX transmission electron microscope (TEM; JEOL, Tokyo, Japan). The size distribution of Exo was measured with a NanoSight nanoparticle tracking analyzer (NS300, Malvern Instruments, Malvern, UK). Additionally, the expression patterns of Exo-specific surface markers (CD63, CD9, and Calnexin) were detected by means of western blot analysis.

### 2.5. Uptake of Exo by MCF-7/ADR

The purified Exo were incubated with PKH26 (Sigma-Aldrich, St Louis, MO) for 5 min, rinsed with PBS, centrifuged at 120000 × *g* for 90 min, resuspended in basal medium, and then incubated with the target cells for 12 h at 37°C. Following incubation, the cells were rinsed twice with PBS and added with 4′,6-diamidino-2-phenylindole (DAPI) (Sigma-Aldrich) to stain the nucleus for 10 min. F-actin was stained with the help of phalloidin-fluorescein isothiocyanate (FITC). Afterwards, the stained cells were observed under an IX53 fluorescence microscope (Olympus, Tokyo, Japan).

### 2.6. Cell Treatment

Based on the known sequences of MALAT1, miR-1-3p, and VASP in the NCBI library, Sangon (Shanghai, China) was commissioned to construct the following plasmids and small interfering RNAs (siRNAs): si-negative control (NC), overexpression (oe)-NC, si-MALAT1, oe-MALAT1, oe-VASP, si-VASP, NC mimic, NC inhibitor, miR-1-3p mimic, and miR-1-3p inhibitor. Additionally, siRNA and overexpression vectors were constructed using pGPU6/Neo (GenePharma Co., Ltd., Shanghai, China) and pCMV6-AC-GFP (Fenghui Biological, Hunan, China) vectors, respectively [19]. MCF-7/S and MCF-7/ADR at passage 3 were transfected using the Lipofectamine 2000 reagent (11668-019, Invitrogen Inc., Carlsbad, CA). Specifically, MCF-7/S cells were manipulated with oe-NC, oe-MALAT1, Exo-oe-NC + ADR, Exo-oe-MALAT1 + ADR, NC inhibitor, miR-1-3p inhibitor, NC inhibitor + si-NC + ADR, miR-1-3p inhibitor + si-NC + ADR, miR-1-3p inhibitor + si-VASP + ADR, oe-VASP, dimethyl sulfoxide (DMSO), and 8-pCPT-2′-O-Me-cAMP (Rap1 specific activator, C8988, Sigma-Aldrich), while MCF-7/ADR cells were subjected to treatment with si-NC, si-MALAT1, Exo-si-NC + ADR, Exo-si-MALAT1 + ADR, NC mimic, miR-1-3p mimic, NC mimic + oe-NC + ADR, miR-1-3p mimic + oe-NC + ADR, miR-1-3p mimic + oe-VASP + ADR, si-VASP, DMSO, and ESI-09 (Rap1 specific inhibitor, 263707-16-0, BioLog Life Science Institute, Germany). The concentration of ADR treatment for MCF-7/S and MCF-7/ADR was set to 0.94 *μ*M and 42.93 *μ*M, respectively, for 48 h. The concentration of 8-pCPT-2′-O-Me-cAMP was set to 100 *μ*M, with 30 min of treatment and that of ESI-09 was set to 10 *μ*M, with 30 min of treatment.

### 2.7. Lentiviral Transduction

Lv-MALAT1 recombinant lentivirus and Lv-NC lentivirus (GenePharma) were prepared and titrated to 10^9^ TU/mL. MCF-7/ADR cells were seeded in a 6-well plate (at a density of 2 × 10^5^ cells/well), and cultured in a humidified incubator for 24 h. MCF-7/ADR cells were transduced with Lv-MALAT1 and Lv-NC. After 72 h, the medium was replaced with a medium containing 4 *μ*g/mL puromycin, and MCF-7/ADR cells were subjected to at least 14-day period of culturing. The amplification of the puromycin-resistant cells was carried out in a medium containing 2 *μ*g/mL puromycin for a total of 9 days. Afterwards, stable MCF-7/ADR cells after overexpressing MALAT1 were obtained in a puromycin-free medium. The obtained Exo were extracted for subsequent animal experimentation.

### 2.8. CCK-8 Assay

MCF-7/S and MCF-7/ADR cells were seeded in a 96-well plate (at a density of 8 × 10^3^ cells/well) and cultured in a humidified incubator for 24 h. Next, the two cell lines were treated with varying concentrations of ADR (MCF-7/S cells: 0, 0.5, 1, 1.5, 2, and 2.5 *μ*mol/L; MCF-7/ADR cells: 0, 20, 40, 60, 80, and 100 *μ*mol/L). After 48 h, 10 *μ*L of CCK-8 solution (Sigma-Aldrich) was supplemented for further culture in a humidified incubator at 37°C for 1 h. Subsequently, the optical density values were detected in each sample at 450 nm with the help of an Epoch microplate spectrophotometer (Bio-Tek, Winooski, VT, USA), and the half maximal inhibitory concentration (IC50) of the two cells was calculated, with 6 repeated wells set for each group.

### 2.9. Scratch Assay

Upon reaching confluence, the cells were scraped with 200-*μ*L tips, rinsed with PBS and cultured with FBS-free RPMI 1640 medium for 24 h. The following day, the cells were photographed at the beginning and end of the experiment using an inverted microscope.

### 2.10. Transwell Assay

Transwell invasion assay was carried out with 50 *μ*L Matrigel (354234, BD Biosciences, Franklin Lakes, NJ, USA) [20]. Photographing was performed using an inverted microscope (IXplore Pro, Olympus) in five randomly selected visual fields, with the number of cells counted.

### 2.11. Luciferase Assay

The DNA fragment containing miR-1-3p, miR-96-5p, miR-101-3p, and miR-1271-5p sequences was respectively inserted into the pmirGLO Dual-Luciferase miRNA Target Expression vector (Promega, Madison, WI, USA) to generate pGL3-basic-MALAT1-Wild Type (MALAT1-WT). Simultaneously, the target-site mutation pGL3-MALAT1-mutant (MALAT1-MUT, sequence: 5′-AUCAGUGACAAGAACUGUCAUA-3′) was constructed. Similarly, VASP 3′-untranslated region (3′-UTR) sequence containing the miR-1-3p-binding site was inserted to obtain VASP-3′-UTR-WT. Afterwards, the target-site mutation VASP-3′-UTR-MUT (sequence: 5′-AAGGAGGGAAUCCCGCGCCAAC-3′) was constructed. The aforementioned plasmids were then co-transfected with NC mimic and miR-1-3p mimic into HEK-293 cells for 24 h. The following day, luciferase activity determination was carried out with a Promega's Glomax 20/20 luminometer (E5311, Shaanxi Zhongmei Biotechnology Co., Ltd., Shaanxi, China).

### 2.12. RNA-Binding Protein Immunoprecipitation (RIP)

The binding between MALAT1, miR-1-3p, and Ago2 protein was determined [21] with 5 *μ*g of rabbit monoclonal antibody to Ago2 (ab186733, dilution ratio of 1 : 50, Abcam Inc., Cambridge, UK) or control IgG antibody (ab172730, dilution ratio of 1 : 100, Abcam). Subsequently, total RNA content was obtained for reverse transcription quantitative polymerase chain reaction (RT-qPCR) detection.

### 2.13. RNA Isolation and Quantitation

Total RNA content was extracted using the TRIzol reagent (15596026, Invitrogen), and then reverse-transcribed into cDNA utilizing PrimeScript Reverse Transcription Reagent kits (RR047A, Takara, Japan). For miRNA detection, the extracted RNA was reverse-transcribed into cDNA with the help of a miRNA-specific stem-loop RT primer. RT-qPCR was subsequently carried out using Fast SYBR Green PCR kits (Applied Biosystems Inc., Carlsbad, CA, USA) on an ABI PRISM 7500 RT-qPCR system (Applied Biosystems). At least 3 repeats were set for each well. U6 was adopted as the loading control for miR-1-3p, and GAPDH for MALAT1 and VASP. The primers are illustrated in Supplementary Table 1. The 2^−ΔΔCt^ method was utilized for quantifying relative expression.

### 2.14. Western Blot Analysis

Western blot analysis was implemented with primary antibodies against VASP (rabbit, dilution ratio of 1 : 1000, #3132, Cell Signaling Technology, Beverly, MA, USA), Rap1 (mouse, dilution ratio of 1 : 100–1 : 1000, sc-53434, Santa Cruz Biotechnology, Inc., CA, USA), and GAPDH (rabbit, dilution ratio of 1 : 1000, #2118, Cell Signaling Technology, serving as the loading control) as well as the secondary antibody goat anti-mouse or goat anti-rabbit IgG (dilution ratio of 1 : 10000, BA1056, Boster Biological Technology Co., Ltd., Wuhan, Hubei, China) labeled with horseradish peroxidase. Subsequently, visualization was carried out with the help of an enhanced chemiluminescence reagent (Thermo Fisher Scientific) on the Amersham Imager 600 (Amersham, Little Chalfont, UK). The ImageJ software was utilized for band intensity quantification.

### 2.15. Rap1-GTPase Pull-Down

In accordance with the manufacturer's instructions of the Active Rap1 Pull-down detection kit (16120, Thermo Fisher Scientific), GST-labeled fusion protein (corresponding to amino acids 788–884 of the human Ral GDS-RAP binding domain bound to glutathione sepharose) was adopted to evaluate the activation of Rap1. The expression patterns of activated small GTPases bound to the magnetic beads were analyzed by western blot analysis and normalized to the total Rap1 expression.

### 2.16. Xenograft Tumor in Nude Mice

A total of 40 female BALB/c nude mice (aged 4–5 weeks, weighing 18–22 g, procured from SJA Laboratory Animal Co., Ltd., Shanghai, China) were housed in a specific pathogen-free environment, with temperature conditions of (25–27)°C and 45%–50% humidity for 1 week, under a 12-h light/dark cycle. Prior to administration, the aforementioned mice were fasted for 12 h and allowed *ad libitum* access to water at other times. MCF-7/S cells treated with Lv-NC and Lv-MALAT1 were injected *in situ* into the inguinal cream pad of nude mice at a density of 5 × 10^6^ cells/0.1 mL. After 1 week, MCF-7/ADR cells treated with Exo-Lv-NC + ADR (100 *μ*g [100 *μ*L PBS] Exo from the Lv-NC-transduced MCF-7/ADR) and Exo-Lv-MALAT1 + ADR (100 *μ*g [100 *μ*L PBS] Exo from the Lv-MALAT1-transduced MCF-7/ADR) were injected into the mice *via* tail vein. Administration was carried out twice a week. The mice following each treatment were intraperitoneally injected with 0.1 mL ADR (25 mg/kg) [22], and administered once every 3 days. The body weight of mice was determined prior to each administration with tumor volume checked every week. Afterwards, the mice were euthanized 8 weeks later, followed by tumor weighing and photographing. The liver and lung tissues of nude mice in each group were collected and fixed with 4% paraformaldehyde for subsequent analysis.

### 2.17. Hematoxylin-Eosin (HE) Staining

The obtained liver and lung tissues were fixed with 4% paraformaldehyde, paraffin-embedded, and sectioned at a thickness of 5 *μ*m. Subsequently, the paraffinized sections were dewaxed, hydrated, and stained with hematoxylin (Beijing Solarbio Science & Technology Co., Ltd., Beijing, China) for 2 min, after which the pathological changes were observed using an optical microscope (XP-330, Shanghai Bingyu Optical Instrument Co., Ltd., Shanghai, China).

### 2.18. Statistical Analysis

The SPSS 22.0 statistical software (IBM Corp., Armonk, NY, USA) and GraphPad Prism 7.0 software were adopted for data analyses. Measurement data were presented as mean ± standard deviation. Unpaired *t*-test, one-way analysis of variance (ANOVA), and Tukey's multiple comparisons test as well as two-way ANOVA or repeated measures ANOVA, followed by Bonferroni post hoc test were utilized for data comparison. A value of *p* < 0.05 was regarded statistically significant.

## 3. Results

### 3.1. MALAT1 Is Highly Expressed in BC Cells and Their Exo

**Figure 1:**
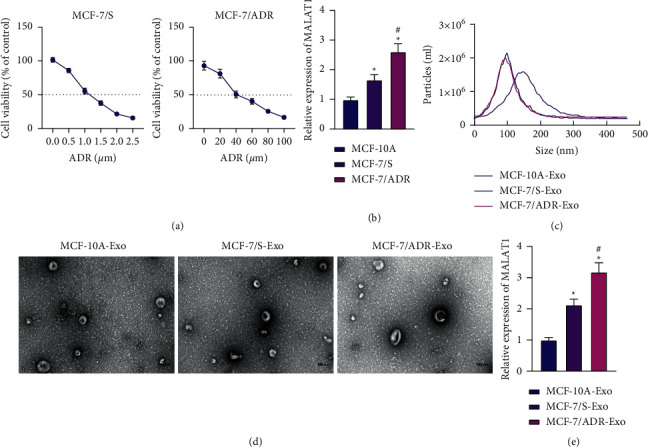
MALAT1 is increased in BC cells and the derived Exo. (a) CCK-8 detection of the viability of MCF-7/S and MCF-7/ADR cells treated with ADR of different concentrations for 48 h. (b) RT-qPCR detection of MALAT1 mRNA expression patterns in MCF-7/S, MCF-7/ADR, and MCF-10A cells. (c) The size distribution of Exo analyzed by NTA. (d) Morphological characterization of the Exo observed using TEM (100 nm). (e) RT-qPCR detection of MALAT1 mRNA expression patterns in MCF-10A-Exo, MCF-7/S-Exo, and MCF-7/ADR-Exo. In panels a and b, ^*∗*^*p* < 0.05, compared with MCF-10A. ^#^*p* < 0.05, compared with MCF-7/S. In panel (e) ^*∗*^*p* < 0.05, compared with MCF-10A-Exo. ^#^*p* < 0.05, compared with MCF-7/S-Exo.

Firstly, we documented a decline of ADR-sensitive and resistant BC cell viability after 48 h of ADR treatment, in a concentration-dependent manner. The IC50 values of MCF-7/S and MCF-7/ADR cells were calculated to be 1.12 *μ*M and 43.10 *μ*M, respectively (Figure 1(a)). The expression levels of MALAT1 were higher in MCF-7/S and MCF-7/ADR cells compared to those in MCF-10A cells, while MCF-7/ADR cells exhibited a more pronounced increase in MALAT1 expression than that in MCF-7/S cells (Figure 1(b)).

Furthermore, the results of TEM and NTA on the isolated Exo illustrated that the particle size ranged between 60 and 200 nm (Figures 1(c) and 1(d)). Meanwhile, Exo markers CD63 and CD9 were both expressed in Exo, while Calnexin was barely expressed (Supplementary Figure 1(a)). As shown in Supplementary Figure 1(b), PKH26-labeled Exo exhibited red fluorescence in the cytoplasm of MCF-7/ADR cells, indicating that the Exo could be internalized by MCF-7/ADR cells.

Additionally, the results of RT-qPCR detection demonstrated an enhancement in MALAT1 expression levels in MCF-7/S-Exo and MCF-7/ADR-Exo (MCF-7/ADR-Exo > MCF-7/S-Exo) (Figure 1(e)). Cumulatively, these findings indicated that MALAT1 may be upregulated in BC cells and the derived Exo, suggesting that MALAT1 could be potentially related to ADR resistance in BC.

### 3.2. BC Cell-Derived Exo Carrying MALAT1 Promote BC Cell Metastasis and Chemoresistance

**Figure 2:**
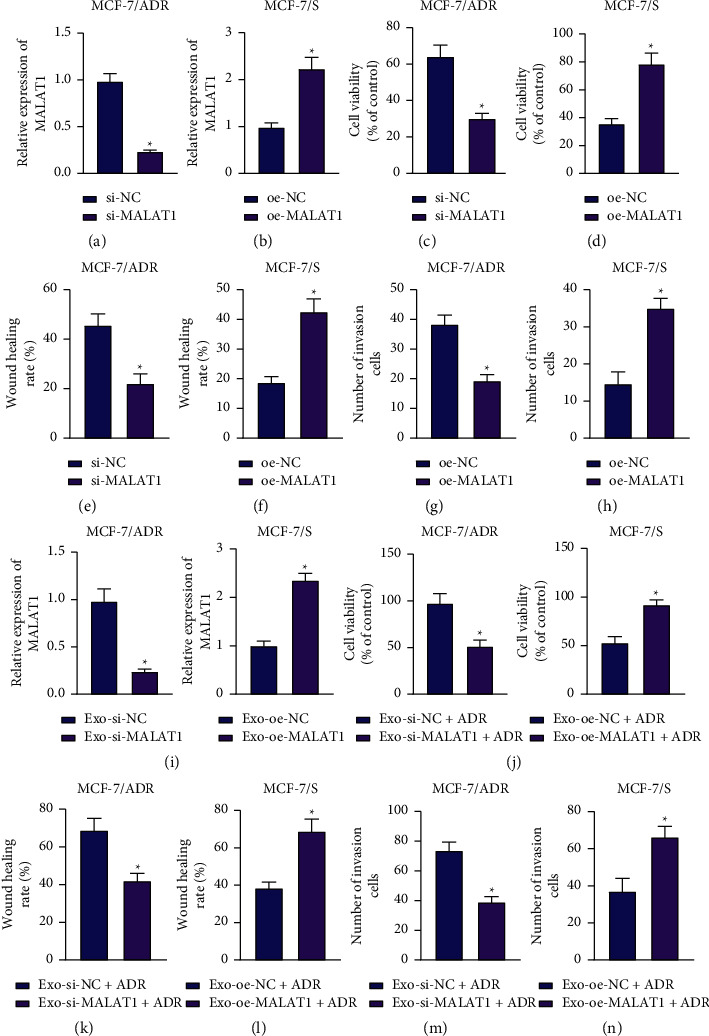
MALAT1 encapsulated by BC cell-derived Exo facilitates BC cell proliferation, metastasis, and chemoresistance. (a) RT-qPCR detection of MALAT1 expression patterns in MCF-7/ADR cells with MALAT1 silencing. (b) RT-qPCR detection of MALAT1 expression patterns in MCF-7/S cells with MALAT1 overexpression. (c) CCK-8 detection of the viability of MCF-7/ADR cells with MALAT1 silencing. (d) CCK-8 detection of the viability of MCF-7/S cells with MALAT1 overexpression. (e) Scratch assay of migration of MCF-7/ADR cells with MALAT1 silencing. (f) Scratch assay of migration of MCF-7/S cells with MALAT1 overexpression. (g) Transwell assay detection of invasion of MCF-7/ADR cells with MALAT1 silencing. (h) Transwell assay detection of invasion of MCF-7/S cells with MALAT1 overexpression. (i) RT-qPCR detection of MALAT1 mRNA expression patterns in MCF-7/S cells treated with Exo-si-MALAT1 or Exo-oe-MALAT1. (j) CCK-8 detection of the viability of MCF-7/S cells treated with Exo-si-MALAT1 + ADR or Exo-oe-MALAT1 + ADR. (k) Scratch assay of migration of MCF-7/S cells treated with Exo-si-MALAT1 + ADR. (l) Scratch assay of migration of MCF-7/S cells treated with Exo-oe-MALAT1 + ADR. (m) Transwell assay detection of invasion of MCF-7/S cells treated with Exo-si-MALAT1 + ADR. (n) Transwell assay detection of invasion of MCF-7/S cells treated with Exo-oe-MALAT1 + ADR. In panels (a–h), ^*∗*^*p* < 0.05, compared with MCF-7/S cells treated with si-NC or oe-NC. In panel (i) ^*∗*^*p* < 0.05, compared with MCF-7/S cells treated with Exo-si-NC or Exo-oe-NC. In panels j–n, ^*∗*^*p* < 0.05, compared with MCF-7/S cells treated with Exo-oe-NC + ADR or Exo-si-NC + ADR. Data are shown as mean ± standard deviation of three technical replicates. Data between two groups were analyzed by unpaired *t*-test.

We then focused on the effect of MALAT1 on BC cell metastasis and chemoresistance. Subsequent experimentation revealed that there was a reduction in MALAT1 expressions levels in MCF-7/ADR cells following MALAT1 silencing, while the opposing trends were documented in MCF-7/S cells overexpressing MALAT1 (Figures 2(a) and 2(b)).

As illustrated in Figures 2(c)–2(h), Supplementary Figures 2(a)–2(d), proliferative, migrative, and invasive abilities of MCF-7/ADR cells were all inhibited in the absence of MALAT1, while the opposite was true for MCF-7/S cells overexpressing MALAT1.

Additionally, the results of RT-qPCR illustrated the downregulation of MALAT1 expression in response to Exo-si-MALAT1 treatment, whereas MALAT1 expression levels were augmented following Exo-oe-MALAT1 treatment (Figure 2(i)). In addition, proliferative, migrative, and invasive abilities of MCF-7/S cells were all diminished in the presence of Exo-si-MALAT1 + ADR, while this effect was negated by Exo-oe-MALAT1 + ADR treatment (Figures 2(j)–2(n), Supplementary Figures 2(e)–2(h)). Together, these findings indicated that BC cell-derived Exo carrying MALAT1 could induce BC cell proliferation, metastasis, and chemoresistance.

### 3.3. MALAT1 Binds to miR-1-3p and Reduces Its Expression in BC Cells

**Figure 3:**
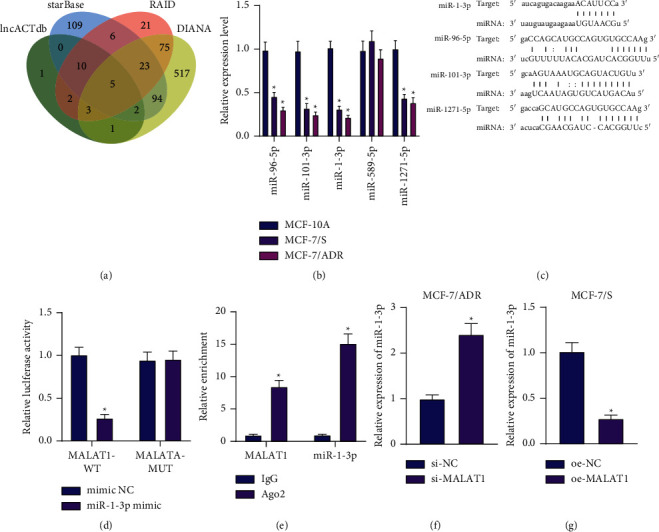
MALAT1 binds to miR-1-3p and consequently decreases its expression in BC cells. (a) Venn diagram of the predicted downstream miRNAs of MALAT1 by the lncACTdb, starBase RAID, and DIANA databases. The four ellipses in the figure represent the prediction results of the four databases, and the central part represents the intersection of the four datasets. (b) RT-qPCR detection of candidate miRNA expression patterns in BC cells. (c) The predicted binding sites between MALAT1 and miR-1-3p, miR-96-5p, miR-101-3p, or miR-1271-5p by the starBase database. (d) Binding between MALAT1 and miR-1-3p confirmed by dual-luciferase reporter gene assay in HEK-293 cells. (e) Enrichment of MALAT1 and miR-1-3p determined by RIP assay. (f) RT-qPCR detection of miR-1-3p expression patterns in MCF-7/ADR cells with silencing of MALAT1. (g) RT-qPCR detection of miR-1-3p expression patterns in MCF-7/S cells overexpressing MALAT1. ^*∗*^*p* < 0.05, compared with MCF-10A, MCF-7/ADR cells treated with si-NC, MCF-7/S cells treated with oe-NC, HEK-293 cells transfected with mimic NC or IgG. Data are shown as mean ± standard deviation of three technical replicates. Data between two groups were analyzed by unpaired *t*-test.

Additionally, we investigated the downstream mechanism of MALAT1 in regard to BC cell proliferation, metastasis, and chemoresistance. The intersection analysis of the predicted downstream miRNAs of MALAT1 highlighted a total of five candidate miRNAs, namely hsa-miR-96-5p, hsa-miR-101-3p, hsa-miR-1-3p, hsa-miR-589-5p, and hsa-miR-1271-5p (Figure 3(a)). Subsequent results of RT-qPCR illustrated that miR-1-3p was remarkably downregulated in BC cells (Figure 3(b)). Moreover, luciferase assay indicated that hsa-miR-96-5p could not bind to MALAT1, while hsa-miR-101-3p and hsa-miR-1271-5p were capable of specifically binding to MALAT1 (Supplementary Figure 3). The implication of the relationship between hsa-miR-101-3p and MALAT1 as well as that between hsa-miR-1271-5p and MALAT1 in chemotherapy resistance of malignant cells has been explored in previous studies [23, 24]; however, the association between has-miR-1-3p and chemotherapy resistance of BC cells remains understudied. Accordingly, has-miR-1-3p was selected as our focus for subsequent experimentation.

Thereafter, binding sites were predicted between MALAT1 and miR-1-3p, miR-96-5p, miR-101-3p, or miR-1271-5p using the starBase database (Figure 3(c)). Further validation by luciferase assay results illustrated that miR-1-3p mimic reduced the luciferase activity of MALAT1-WT without altering that of MALAT1-MUT (Figure 3(d)). Meanwhile, RIP data displayed a rising trend in the enrichment of both MALAT1 and miR-1-3p in cells incubated with Ago2 antibody (Figure 3(e)). Furthermore, the results of RT-qPCR confirmed the presence of upregulated miR-1-3p expression levels in si-MALAT1-treated MCF-7/ADR cells, while the opposing trends were documented in MCF-7/S cells overexpressing MALAT1 (Figures 3(f) and 3(g)). Altogether, these findings highlighted that MALAT1 was capable of binding to miR-1-3p and inhibiting its expression in BC cells.

### 3.4. miR-1-3p Targets VASP and Inhibits Its Expression in BC Cells

**Figure 4:**
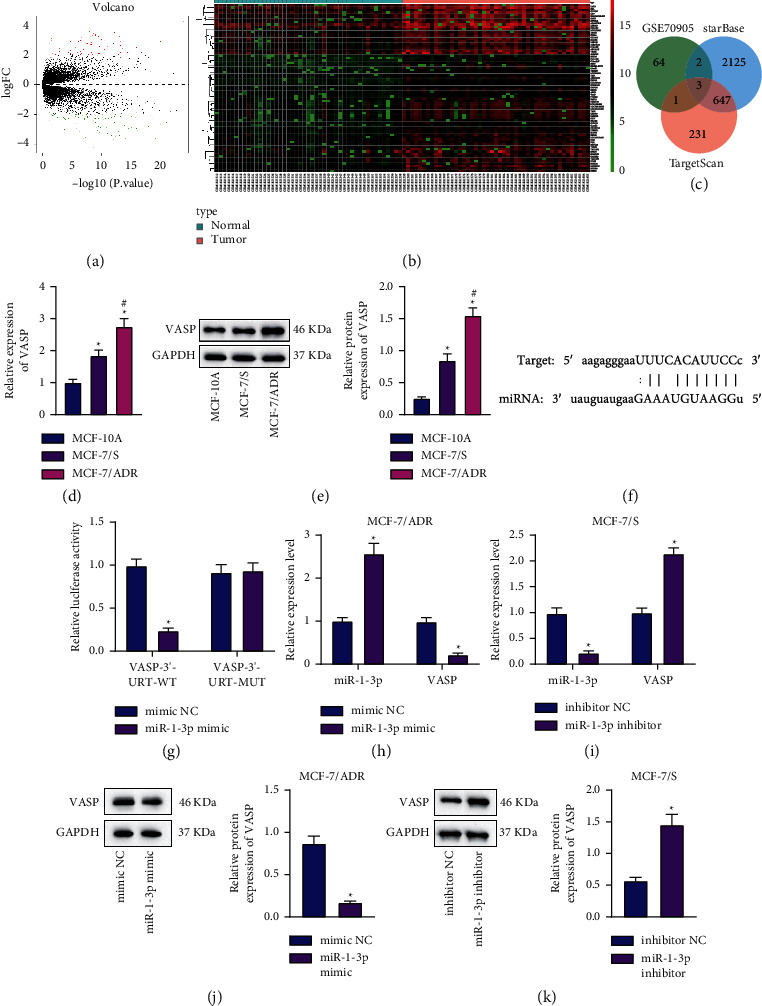
VASP is a target gene of miR-1-3p in BC cells. (a) A volcano plot of differentially expressed genes in BC in the GSE70905 dataset. The abscissa represents −log10 *p*value, and the ordinate represents logFoldChange; the red point represents the significantly highly expressed gene in the tumor, and the green point represents the significantly poorly expressed gene. (b) A heat map of significantly upregulated genes in BC in the GSE70905 dataset. The abscissa represents the sample number, and the ordinate represents the gene name; each small square represents the expression of a gene in a sample, and the histogram at the upper right is the color scale. (c) Venn diagram of significantly upregulated genes in BC in the GSE70905 dataset and the predicted miR-1-3p target genes by the starBase and TargetScan databases. The central part represents the intersection of the three sets of data. (d) RT-qPCR detection of VASP mRNA expression in MCF-7/ADR, MCF-7/S, and MCF-10A cells. ^*∗*^*p* < 0.05, compared with MCF-10A cells. ^#^*p* < 0.05, compared with MCF-7/S cells. (e) Western blot analysis of VASP protein expression patterns in MCF-7/ADR, MCF-7/S, and MCF-10A cells. ^*∗*^*p* < 0.05, compared with MCF-10A cells. ^#^*p* < 0.05, compared with MCF-7/S cells. (f) miR-1-3p binding sites in the 3′-UTR of VASP predicted by the starBase database. (g) miR-1-3p binding to VASP verified by dual-luciferase reporter gene assay in HEK-293 cells. ^*∗*^*p* < 0.05, compared with HEK-293 cells transfected with NC mimic. (h) RT-qPCR detection of miR-1-3p and VASP expression in MCF-7/ADR cells treated with miR-1-3p mimic. ^*∗*^*p* < 0.05, compared with MCF-7/ADR cells transfected with NC mimic. (i) RT-qPCR detection of miR-1-3p and VASP expression in MCF-7/S cells treated with miR-1-3p inhibitor. ^*∗*^*p* < 0.05, compared with MCF-7/S cells treated with NC inhibitor. (j) Western blot analysis of VASP protein expression patterns in MCF-7/ADR cells treated with miR-1-3p mimic. ^*∗*^*p* < 0.05, compared with MCF-7/ADR treated with NC mimic. (k) Western blot analysis of VASP protein expression patterns in MCF-7/S treated with miR-1-3p inhibitor. ^*∗*^*p* < 0.05, compared with MCF-7/S treated with NC inhibitor. Data are shown as mean ± standard deviation of three technical replicates. Data between two groups were analyzed by unpaired *t*-test.

We extended our approach to mechanistic findings to determine the mechanism of miR-1-3p underlying the control of BC development. Differential analyses of the BC-related GSE70905 dataset reared a total of 166 differentially expressed genes in BC, wherein 70 genes were highly expressed in BC tissues (Figures 4(a) and 4(b)). Subsequently, these highly expressed genes were intersected with the miR-1-3p target genes predicted by the starBase and TargetScan databases, and the results highlighted 3 candidate genes (Figure 4(c)). Compared with normal samples, the expression of VASP in BC samples was found to be the most profoundly upregulated among these three genes (Supplementary Table 2).

Furthermore, there were enhanced VASP expressions in MCF-7/ADR and MCF-7/S cells compared to those in MCF-10A cells, wherein MCF-7/ADR cells exhibited the most prominent increase in VASP expression levels (Figures 4(d) and 4(e)). Moreover, the starBase database predicted the presence of miR-1-3p binding sites in the 3′-UTR of VASP (Figure 4(f)), which was further verified by subsequent luciferase assay results (Figure 4(g)). In addition, the expression levels of miR-1-3p were enhanced in the MCF-7/ADR cells treated with miR-1-3p mimic, whereas those of VASP were diminished. In contrast, in MCF-7/S cells, miR-1-3p inhibitor reduced the miR-1-3p expression, while upregulating that of VASP (Figures 4(h)–4(k)). Together, these findings indicated that miR-1-3p was capable of targeting VASP and suppressing its expression in BC cells.

### 3.5. miR-1-3p Reduces BC Cell Metastasis and Chemoresistance by Limiting VASP

**Figure 5:**
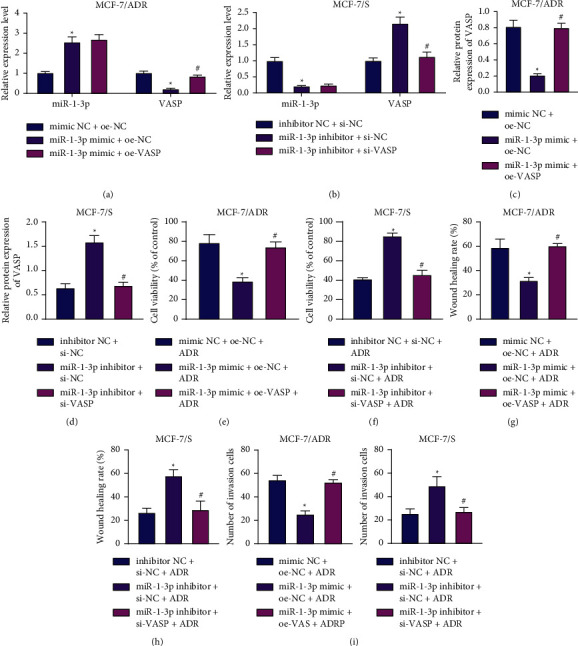
miR-1-3p-mediated VASP inhibition reduces BC cell metastasis and chemoresistance. (a) RT-qPCR detection of miR-1-3p and VASP expression patterns in MCF-7/ADR cells treated with miR-1-3p mimic or combined with oe-VASP. ^*∗*^*p* < 0.05, compared with MCF-7/ADR cells treated with mimic + oe-NC. ^#^*p* < 0.05, compared with MCF-7/ADR cells treated with miR-1-3p mimic + oe-NC. (b) RT-qPCR detection of miR-1-3p and VASP expression patterns in MCF-7/S cells treated with miR-1-3p inhibitor or combined with si-VASP. ^*∗*^*p* < 0.05, compared with MCF-7/S cells treated with NC inhibitor + si-NC. ^#^*p* < 0.05, compared with MCF-7/S cells treated with miR-1-3p inhibitor + si-NC. (c) Western blot analysis of VASP protein expression patterns in MCF-7/ADR cells treated with miR-1-3p mimic or combined with oe-VASP. ^*∗*^*p* < 0.05, compared with MCF-7/ADR cells treated with NC mimic + oe-NC. ^#^*p* < 0.05, compared with MCF-7/ADR cells treated with miR-1-3p mimic + oe-NC. (d) Western blot analysis of VASP protein expression patterns in MCF-7/S cells treated with miR-1-3p inhibitor or combined with si-VASP. ^*∗*^*p* < 0.05, compared with MCF-7/S cells treated with NC inhibitor + si-NC. ^#^*p* < 0.05, compared with MCF-7/S cells treated with miR-1-3p inhibitor + si-NC. (e) CCK-8 detection of the viability of MCF-7/ADR cells treated with miR-1-3p mimic or combined with oe-VASP. (f) CCK-8 detection of the viability of MCF-7/S cells treated with miR-1-3p inhibitor or combined with si-VASP. (g) Scratch assay of migration of MCF-7/ADR cells treated with miR-1-3p mimic or combined with oe-VASP. (h) Scratch assay of migration of MCF-7/S cells treated with miR-1-3p inhibitor or combined with si-VASP. (i) Transwell assay detection of invasion of MCF-7/ADR cells treated with miR-1-3p mimic or combined with oe-VASP. (j)Transwell assay detection of invasion of MCF-7/S cells treated with miR-1-3p inhibitor or combined with si-VASP. In panels (e), (g), and (i) ^*∗*^*p* < 0.05, compared with MCF-7/ADR cells treated with NC mimic + oe-NC + ADR. ^#^*p* < 0.05, compared with MCF-7/ADR cells treated with miR-1-3p mimic + oe-NC + ADR. In panels (f), (h), and (j) ^*∗*^*p* < 0.05, compared with MCF-7/S cells treated with NC inhibitor + si-NC + ADR. ^#^*p* < 0.05, compared with MCF-7/S cells treated with miR-1-3p inhibitor + si-NC + ADR. Data are shown as mean ± standard deviation of three technical replicates.

We further explored the significance of miR-1-3p in BC cell metastasis and chemoresistance *via* modulation of VASP expression. We documented an enhancement in miR-1-3p expression levels, but a reduction in those of VASP in MCF-7/ADR cells following miR-1-3p overexpression, whereas these trends were reversed by further overexpression of VASP. In MCF-7/S cells, there was a decline in the miR-1-3p expression, yet an increase in VASP expression in the absence of miR-1-3p. Meanwhile, combined treatment with miR-1-3p inhibitor and si-VASP led to inhibition of VASP expression (Figures 5(a)–5(d), Supplementary Figures 4(a) and 4(b)).

The results displayed in Figures 5(e)–5(j) and Supplementary Figures 4(c) and 4(c) illustrated that treatment with miR-1-3p mimic + oe-NC + ADR impaired the proliferative, migrative, and invasive abilities of MCF-7/ADR cells, while these effects were undermined by miR-1-3p mimic + oe-VASP + ADR treatment. Conversely, in MCF-7/S cells, the above abilities were all enhanced in the presence of miR-1-3p inhibitor + si-NC + ADR, while treatment with miR-1-3p inhibitor + si-VASP + ADR led to opposite results. Altogether, these findings indicated that miR-1-3p could affect BC cell metastasis and chemoresistance by downregualting VASP expression.

### 3.6. Silencing of VASP Blocks the Rap1 Signaling Pathway in BC Cells

**Figure 6:**
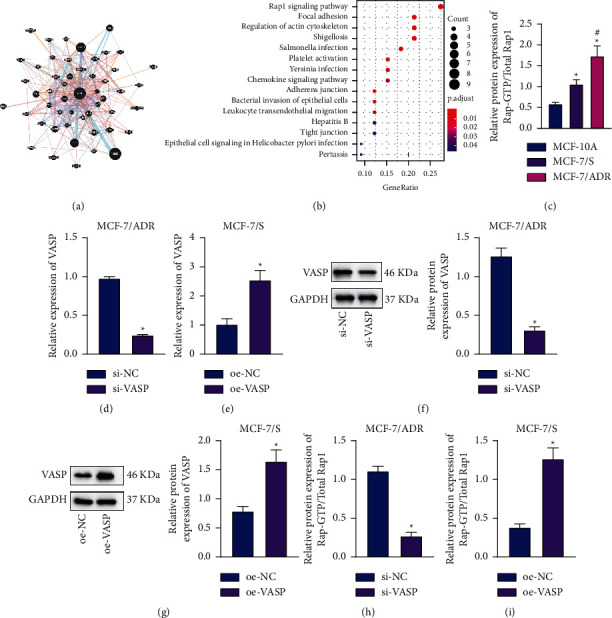
Silencing of VASP inactivates the Rap1 signaling pathway in BC cells. (a) VASP-related gene interaction network. Each circle represents a gene, and the lines between the circles indicate the correlation between genes. (b) KEGG enrichment analysis of VASP-related genes. The abscissa represents GeneRatio, and the ordinate indicates KEGG entry identifier; each circle represents the number of genes enriched in an entry identifier, and the histogram on the right is the color scale. (c) Western blot analysis of Rap1-GTP/total Rap1 ratio in MCF-7/ADR, MCF-7/S, and MCF-10A cells. ^*∗*^*p* < 0.05, compared with MCF-10A cells. ^#^*p* < 0.05, compared with MCF-7/S cells. (d) RT-qPCR detection of VASP expression patterns in MCF-7/ADR cells with si-VASP. (e) RT-qPCR detection of VASP expression patterns in MCF-7/S cells with overexpression of VASP. (f) Western blot analysis of VASP protein expression patterns in MCF-7/ADR cells with si-VASP. (g) Western blot analysis of VASP protein expression patterns in MCF-7/S cells with overexpression of VASP. (h) Western blot analysis of Rap1-GTP/total Rap1 ratio in MCF-7/ADR cells with si-VASP. (i) Western blot analysis of Rap1-GTP/total Rap1 ratio in MCF-7/S cells with overexpression of VASP. In panels (d), (f), and (h) ^*∗*^*p* < 0.05, compared with MCF-7/ADR treated with si-NC. In panels (e), (g), and (i) ^*∗*^*p* < 0.05, compared with MCF-7/S treated with oe-NC. Data are shown as mean ± standard deviation of three technical replicates.

Thereafter, we explored the downstream factors of VASP. The GeneMANIA database identified a total of 50 VASP-related genes (Figure 6(a)), and subsequent KEGG enrichment analysis of these genes suggested that these genes were primarily enriched in the Rap1 signaling pathway (Figure 6(b)).

Further results of western blot analysis illustrated higher Rap1-GTP/total Rap1 ratio in MCF-7/ADR and MCF-7/S cells compared to that in MCF-10A cells, with a more profound increase in MCF-7/ADR cells (Figure 6(c)). The latter finding unfolded that Rap1 was indeed activated in BC cells in relation to BC chemoresistance.

We further documented a reduction in VASP mRNA expression levels in MCF-7/ADR cells following silencing of VASP, while MCF-7/S cells overexpressing VASP exhibited increased VASP mRNA expression (Figures 6(d) and 6(e)). Additionally, silencing of VASP in MCF-7/ADR cells suppressed the protein expression of VASP and Rap1-GTP/total Rap1 ratio, whereas the overexpression of VASP in MCF-7/S cells led to the opposite trends (Figures 6(f)–6(i)). Overall, these findings indicated that silencing of VASP was capable of blocking the Rap1 signaling pathway in BC cells.

### 3.7. Inhibition of Rap1 Signaling Pathway Reduces BC Cell Metastasis and Chemoresistance

**Figure 7:**
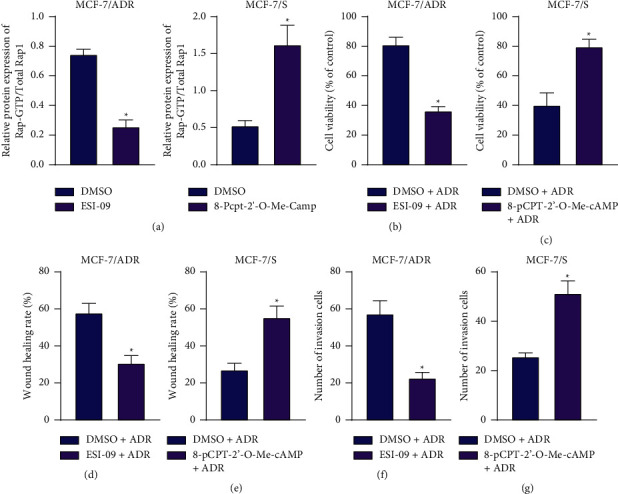
Inactivated Rap1 signaling pathway attenuates BC cell proliferation, metastasis, and chemoresistance. (a) Western blot analysis of Rap1-GTP and total Rap1 in the MCF-7/ADR cells treated with ESI-09 and MCF-7/S cells treated with 8-pCPT-2′-O-Me-cAMP. (b) CCK-8 detection of the viability of MCF-7/ADR cells treated with ESI-09 ± ADR. (c) CCK-8 detection of the viability of MCF-7/S cells treated with 8-pCPT-2′-O-Me-cAMP. (d) Scratch assay of migration of MCF-7/ADR cells treated with ESI-09 ± ADR. (e) Scratch assay of migration of MCF-7/S cells treated with 8-pCPT-2′-O-Me-cAMP. (f) Transwell assay detection of invasion of MCF-7/ADR cells treated with ESI-09 ± ADR. (g) Transwell assay detection of invasion of MCF-7/S cells treated with 8-pCPT-2′-O-Me-cAMP. ^*∗*^*p* < 0.05, compared with MCF-7/ADR or MCF-7/S treated with DMSO + ADR. Data are shown as mean ± standard deviation of three technical replicates.

Thereafter, we investigated the significance of the Rap1 signaling pathway in BC cell metastasis and drug resistance. We documented a reduction in Rap1-GTP and total Rap1 expression levels, along with depleted Rap1-GTP/total Rap1 ratio in MCF-7/ADR cells treated with ESI-09. On the other hand, treatment with 8-pCPT-2′-O-Me-cAMP in MCF-7/S led to an enhancement in Rap1-GTP and total Rap1 expressions along with increased Rap1-GTP/total Rap1 ratio (Figure 7(a)).

Subsequent results of functional assays revealed that the proliferative, migrative, and invasive abilities of MCF-7/ADR cells treated with ESI-09 + ADR were all diminished, but opposing trends were documented in MCF-7/S cells treated with 8-pCPT-2′-O-Me-cAMP + ADR (Figures 7(b)–7(g)). Collectively, these findings indicated that inactivation of the Rap1 signaling pathway could impede BC cell proliferation, metastasis, and chemoresistance.

### 3.8. MALAT1 Shuttled by BC Cell-Derived Exo Promotes Tumorigenesis and Metastasis of BC Cells *In Vivo* by Regulating the miR-1-3p/VASP/Rap1 Axis

**Figure 8:**
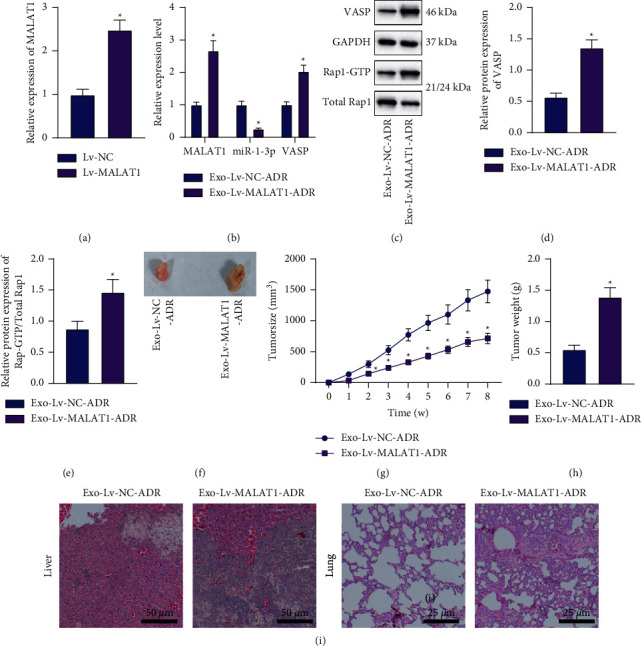
MALAT1 shuttled by BC cell-derived Exo boosts the tumorigenesis and metastasis of BC cells *in vivo* by regulating the miR-1-3p/VASP/Rap1 axis. (a) RT-qPCR detection of MALAT1 expression patterns in tumor tissues of mice injected with Lv-MALAT1-transduced MCF-7/ADR. ^*∗*^*p* < 0.05, compared with mice treated with Lv-NC. (b) RT-qPCR detection of MALAT1, miR-1-3p, and VASP expression patterns in tumor tissues of mice treated with Exo-Lv-MALAT1 + ADR. (c) Western blot analysis of VASP expression patterns and Rap1-GTP/total Rap1 ratio in tumor tissues of mice treated with Exo-Lv-MALAT1 + ADR. (d) Quantitative analysis of VASP expression patterns in tumor tissues of mice treated with Exo-Lv-MALAT1 + ADR. (e) Quantitative analysis of Rap1-GTP/total Rap1 ratio in tumor tissues of mice treated with Exo-Lv-MALAT1 + ADR. (f) Representative images showing xenografts in nude mice treated with Exo-Lv-MALAT1 + ADR. (g) Tumor volume of mice treated with Exo-Lv-MALAT1 + ADR at the 0–8th week. (h) Tumor weight of mice treated with Exo-Lv-MALAT1 + ADR at the 8th week. (i) HE staining analysis of liver metastasis and lung metastasis of BC cells in mice treated with Exo-Lv-MALAT1 + ADR. ^*∗*^*p* < 0.05, compared with mice treated with Exo-Lv-NC + ADR. *n* = 10 for mice following each treatment.

Lastly, we aimed to characterize the effects of MALAT1 shuttled by BC cell-derived Exo *via* the miR-1-3p/VASP/Rap1 axis on the tumorigenesis and metastasis *in vivo*. MALAT1 upregulation was apparent in tumor tissues of mice injected with Lv-MALAT1-transduced MCF-7/ADR cells (Figure 8(a)). In addition, treatment with Exo-Lv-MALAT1 + ADR increased the MALAT1 and VASP mRNA expression levels, while decreasing those of miR-1-3p (Figure 8(b)). Besides, VASP protein expression and Rap1-GTP/total Rap1 ratio were both augmented in tumor tissues of mice treated with Exo-Lv-MALAT1 + ADR (Figures 8(c)–8(e)).

Furthermore, tumor volume and weight were both augmented following treatment with Exo-Lv-MALAT1 + ADR (Figures 8(f)–8(h)). Meanwhile, results of HE staining shown in Figure 8(i) illustrated an enhancement in both liver metastasis and lung metastasis of BC cells. Taken together, the abovementioned evidence indicated that MALAT1 shuttled by BC cell-derived Exo could regulate the miR-1-3p/VASP/Rap1 axis, and thus induce tumorigenesis and metastasis of BC cells *in vivo*.

## 4. Discussion

Exo derived from different cells are known to exhibit a plethora of critical effects in the course of cancer progression [25]. Expanding our knowledge of the same, findings uncovered in our study highlighted the promotive effect of MALAT1 encapsulated by BC cell-derived Exo on BC metastasis and chemoresistance *via* the miR-1-3p/VASP/Rap1 pathway.

Initially, we documented the upregulation of MALAT1 in BC cells and their Exo, while MALAT1 delivered by BC cell-derived Exo brought about augmented BC cell metastasis and chemoresistance. Consistently, abundantly expressed MALAT1 was previously detected in BC cells and Exo, such that Exo-mediated MALAT1 was indicated to be capable of inducing BC cell proliferation [26]. Moreover, a prior study further highlighted the correlation between MALAT1 and tumor-node-metastasis stage, distant metastasis, and survival outcomes of patients, which is also in line with our findings [27]. Further corroborating our data, the study performed by Shaath et al. underscored the potential role of MALAT1 in driving triple-negative BC resistance to neoadjuvant chemotherapy [28]. In lieu of the abovementioned findings, it would be plausible to propose MALAT1 shuttled by BC cell-derived Exo as a potential biomarker for predicting ADR resistance in BC.

Subsequent experimentation in our study unveiled that MALAT1 potentially bound to miR-1-3p and limited its expression in BC cells. The latter does not come as a surprise as there is much evidence indicating the direct binding of MALAT1 to miR-1-3p, wherein MALAT1 could negatively regulate the expression of miR-1-3p in esophagus cancer cells [29]. In addition, the study carried out by Sun et al. evidenced downregulation of miR-1-3p in BC patients with sentinel lymph node metastasis, which is in accordance with our findings [30]. High expression of miR-1-3p was further shown to confer an inhibitory effect on BC cell viability, invasion, and migration, while simultaneously suppressing tumor formation and metastasis [13].

miRNAs are well-established to possess the ability to limit the expressions of target mRNAs *via* interaction with their 3′-UTR [31]. Herein, our findings elaborated on the binding of miR-1-3p to VASP. This particular discovery represents the first evidence for posttranscriptional regulation of VASP by miR-1-3p in BC cells and may be of significant importance for regulating BC progression. Interestingly, the promotion of VASP expression was previously shown to lead to augmented malignant potentials of BC cells [32]. It is also noteworthy that, enforced VASP expression in both MCF-7 and BT-549 cells exerted a countering effect on the promoting action of miR-16-5p on tamoxifen sensitivity [15]. Together, these findings and evidence highlight the ability of miR-1-3p to reduce BC cell metastasis and chemoresistance by limiting VASP. The miR-1-3p-mediated inhibition of VASP could serve as a potential mechanism to suppress cancer progression and enhance the chemotherapy sensitivity of BC cells.

Additional analyses in our study further elaborated that silencing of VASP disrupted the activation of the Rap1 signaling pathway in BC cells. Meanwhile, a prior study indicated that the activity of Rap1 and phosphorylation of VASP are both elevated by G-1 treatment [33], which reiterates a possible positive correlation between Rap1 and VASP. In addition, the study performed by Benz et al. documented a blockage of Rap1b in platelets from VASP-null mice compared to platelets from WT mice, again in accordance with our findings [34]. Furthermore, we also learnt that blockage of the Rap1 signaling pathway led to the arrest of BC cell metastasis and chemoresistance. Similarly, Rap1a is capable of activating the MAPK signaling pathway and thus bring about malignant features of BC cells [35]. Moreover, existing evidence further indicates that RAP1 depletion confers an enhancing effect on chemosensitivity of cancer cells [36, 37]. Meanwhile, MALAT1 was also previously shown to reduce Rap1B by sponging miR-101 in U251 and U87 cells [38]. Altogether, the aforementioned findings underscore the important role of the MALAT1/miR-1-3p/VASP/Rap1 regulatory network in the development and chemoresistance of BC and provide a novel viewpoint for exploring new pathogenesis and biomarkers of BC.

## 5. Conclusion

**Figure 9:**
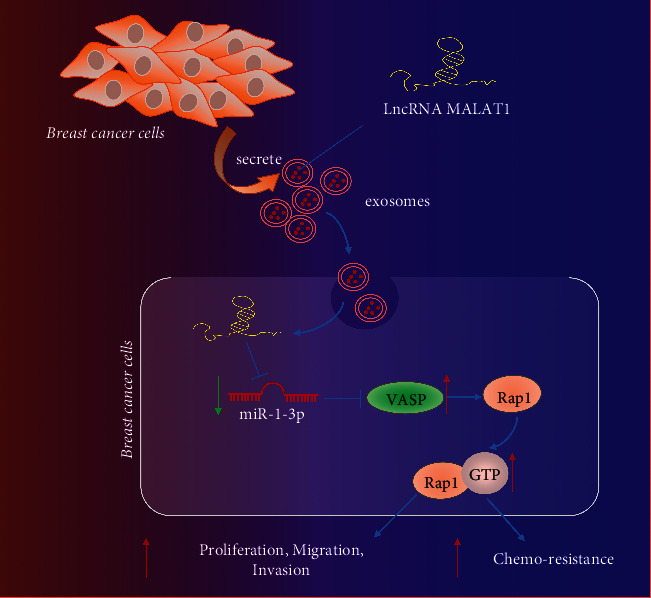
Schematic diagram of the mechanism by which MALAT1 delivered by BC cell-derived Exo affects BC metastasis and chemoresistance. BC cell-derived Exo deliver MALAT1, which downregulates miR-1-3p, and upregulates VASP, thereby activating the Rap1 signaling pathway to promote BC metastasis and chemoresistance.

Collectively, our findings highlight the oncogenic role of MALAT1 mediated by BC cell-derived Exo in BC. The upregulation of MALAT1 by BC cell-derived Exo in BC induces cell metastasis and decreases ADR sensitivity, and the reported mechanism is associated with the miR-1-3p-mediated VASP/Rap1 axis (Figure 9). Overall, we hope our research draws attention to the mechanism of BC cell metastasis and resistance modulated by BC cell-derived Exo MALAT1 regulation and paves the way for novel strategies for overcoming BC resistance.

## Data Availability

The data that support the findings of this study are included in the manuscript and Supplementary Materials.

## Supplementary Materials

Supplementary Materials Supplementary Table 1. Primer sequences for reverse transcription quantitative polymerase chain reaction. Supplementary Table 2. Differential expression of candidate target genes. Supplementary Figure 1(a), Western blot analysis of Exo maker proteins CD63, CD9, and Calnexin in the Exo. (b), Uptake of BC cell-derived Exo by MCF-7/ADR cells (25 *μ*m). Fluorescent phalloidin-FITC (green) was used to stain F-actin, and DAPI (blue) was used to stain the nuclei. Supplementary Figure 2(a), Scratch test of migration of MCF-7/ADR cells with MALAT1 silencing. (b), Scratch test of migration of MCF-7/S cells with MALAT1 overexpression. (c), Transwell assay detection of invasion of MCF-7/ADR cells with MALAT1 silencing (200 ×). (d), Transwell assay detection of invasion of MCF-7/S cells with MALAT1 overexpression (200 ×). (e), Scratch test of migration of MCF-7/ADR cells treated with Exo-si-MALAT1 + ADR. (f), Scratch test of migration of MCF-7/S cells treated with Exo-oe-MALAT1 + ADR. (g), Transwell assay detection of invasion of MCF-7/ADR cells treated with Exo-si-MALAT1 + ADR (200 ×). (h), Transwell assay detection of invasion of MCF-7/S cells treated with Exo-oe-MALAT1 + ADR (200 ×). Supplementary Figure 3. Dual-luciferase reporter gene assay on binding between MALAT1 and hsa-miR-101-3p (a), hsa-miR-96-5p (b), or hsa-miR-1271-5p (c). Supplementary Figure 4(a), Western blot analysis of VASP protein band in MCF-7/ADR cells treated with miR-1-3p mimic or combined with oe-VASP. (b), Western blot analysis of VASP protein in MCF-7/S cells treated with miR-1-3p inhibitor or combined with si-VASP. (c), Transwell assay detection of invasion of MCF-7/ADR cells treated with miR-1-3p mimic or combined with oe-VASP (200 ×). (d), Transwell assay detection of invasion of MCF-7/S cells treated with miR-1-3p inhibitor or combined with si-VASP (200 ×).
