# Supplementary material for: Exosomes Derived from Tumor Cells Initiate Breast Cancer Cell Metastasis and Chemoresistance through a MALAT1-Dependent Mechanism
Source: J Oncol. 2022 Jun 30;2022:5483523. doi: 10.1155/2022/5483523 (PMC9262507; doi:10.1155/2022/5483523)
Supplement: Supplementary Materials — Supplementary Table 1. Primer sequences for reverse transcription quantitative polymerase chain reaction. Supplementary Table 2. Differential expression of candidate target genes. Supplementary Figure 1(a), Western blot analysis of Exo maker proteins CD63, CD9, and Calnexin in the Exo. (b), Uptake of BC cell-derived Exo by MCF-7/ADR cells (25 μm). Fluorescent phalloidin-FITC (green) was used to stain F-actin, and DAPI (blue) was used to stain the nuclei. Supplementary Figure 2(a), Scratch test of migration of MCF-7/ADR cells with MALAT1 silencing. (b), Scratch test of migration of MCF-7/S cells with MALAT1 overexpression. (c), Transwell assay detection of invasion of MCF-7/ADR cells with MALAT1 silencing (200 ×). (d), Transwell assay detection of invasion of MCF-7/S cells with MALAT1 overexpression (200 ×). (e), Scratch test of migration of MCF-7/ADR cells treated with Exo-si-MALAT1 + ADR. (f), Scratch test of migration of MCF-7/S cells treated with Exo-oe-MALAT1 + ADR. (g), Transwell assay detection of invasion of MCF-7/ADR cells treated with Exo-si-MALAT1 + ADR (200 ×). (h), Transwell assay detection of invasion of MCF-7/S cells treated with Exo-oe-MALAT1 + ADR (200 ×). Supplementary Figure 3. Dual-luciferase reporter gene assay on binding between MALAT1 and hsa-miR-101-3p (a), hsa-miR-96-5p (b), or hsa-miR-1271-5p (c). Supplementary Figure 4(a), Western blot analysis of VASP protein band in MCF-7/ADR cells treated with miR-1-3p mimic or combined with oe-VASP. (b), Western blot analysis of VASP protein in MCF-7/S cells treated with miR-1-3p inhibitor or combined with si-VASP. (c), Transwell assay detection of invasion of MCF-7/ADR cells treated with miR-1-3p mimic or combined with oe-VASP (200 ×). (d), Transwell assay detection of invasion of MCF-7/S cells treated with miR-1-3p inhibitor or combined with si-VASP (200 ×). [file 5483523.f1.docx]

**Supplementary Table 1** Primer sequences for reverse transcription quantitative polymerase chain reaction

| Gene | Primer sequences |
| --- | --- |
| MALAT1-H | F: 5′-GAATTGCGTCATTTAAAGCCTAGTT-3′ |
|  | R: 5′-GTTTCATCCTACCACTCCCAATTAAT-3′ |
| VASP-H | F: 5’-CTGGGAGAAGAACAGCACAACC-3 |
|  | R: 5’-AGGTCCGAGTAATCACTGGAGC-3’ |
| GAPDH-H | F: 5′-GAGAAGGCTGGGGCTCATTT-3′ |
|  | R: 5′-AGTGATGGCATGGACTGTGG-3′ |
| miR-1-3p-H | F: 5’-TGCCCATATGGACCTGCTAAG-3′ |
|  | R: reverse universal primer |
| U6-H | F: 5′-CTCGCTTCGGCAGCACA-3′ |
|  | R: 5′-reverse universal primer |
| MALAT1-M | F: 5′-TGCAGTGTGCCAATGTTTCG-3′ |
|  | R: 5’-GGCCAGCTGCAAACATTCAA-3′ |
| VASP-M | F: 5’-TATCTGGGGCAGGTCATGGA-3 |
|  | R: 5’-CTCCTCCTGCTTGCTCACTT-3’ |
| miR-1-3p-M | F: 5’-TGCCTACTCAGAGCACATACT-3’ |
|  | R: reverse universal primer |
| U6-M | F: 5’-CTCGCTTCGGCAGCACA-3′ |
|  | R: reverse universal primer |
| GAPDH -M | F: 5'-TGCACCACCAACTGCTTAGC-3' |
|  | R: 5'-GGCATGGACTGTGGTCATGAG-3' |

Note: F, forward; R, reverse; MALAT1, metastasis associated lung adenocarcinoma transcript 1; VASP, vasodilator-stimulated phosphoprotein; GAPDH, glyceraldehyde-3-phosphate dehydrogenase; miR-1-3p, microRNA-1-3p; H, human; M, mouse.

**Supplementary Table 2** Differential expression of candidate target genes.

| Symbol | logFoldChange | *p* value | adj.*p*.Val |
| --- | --- | --- | --- |
| VASP | 2.04348622 | 2.99E-09 | 1.19E-07 |
| TNKS2 | 2.704185608 | 7.70E-09 | 2.72E-07 |
| ZFP36L2 | 3.149758412 | 1.91E-07 | 4.54E-06 |

Note: symbol: gene name; logFoldChange: the differential expression multiple of the candidate gene in the tumor with normal samples serving as the control; *p* value: the *p* value of the differential gene; adj.*p*.Val: the *p* value corrected by the FDR method; VASP, vasodilator-stimulated phosphoprotein; TNKS2, Tankyrase 2; ZFP36L2, ZFP36 ring finger protein like 2.

**
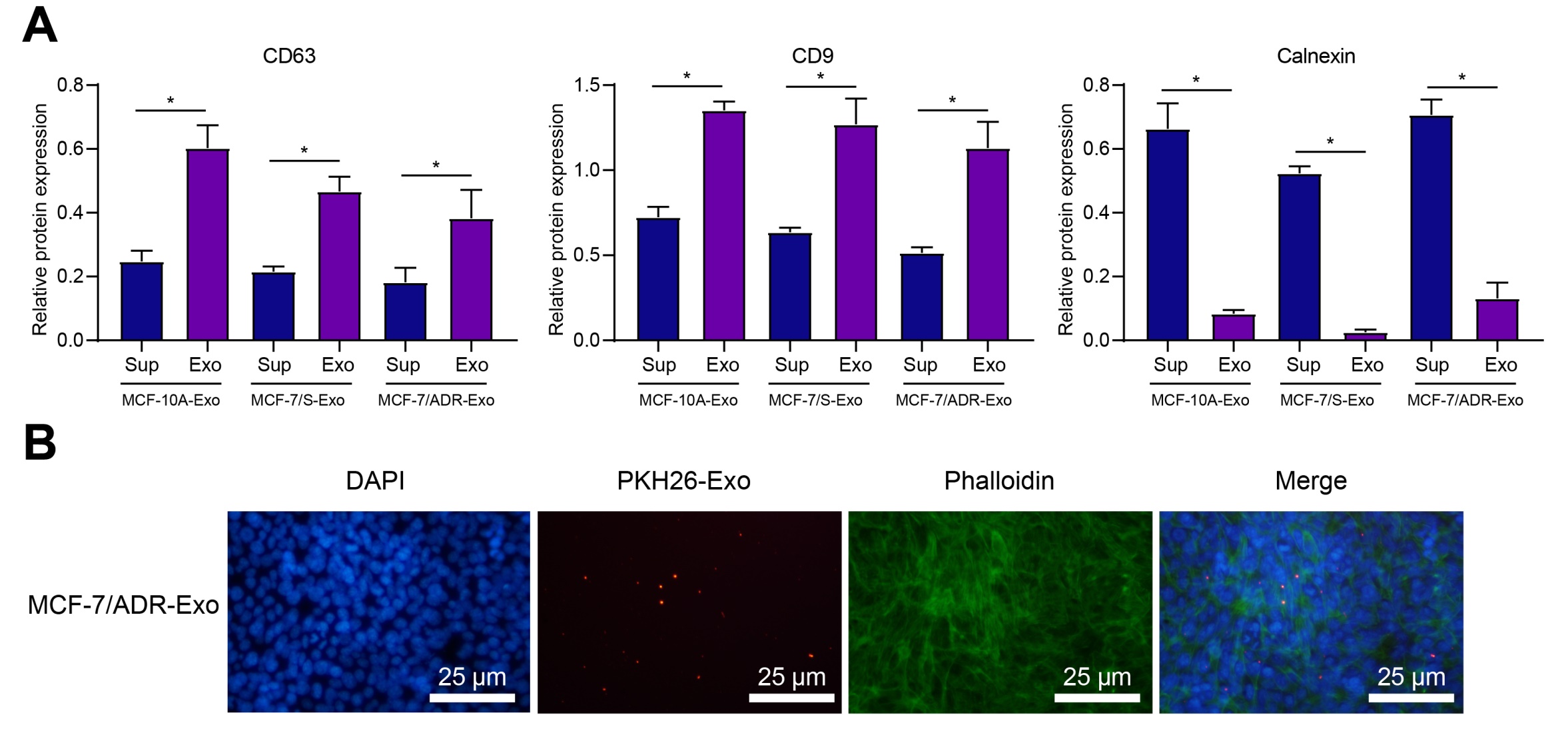
**

**Supplementary Figure 1** A, Western blot analysis of Exo maker proteins CD63, CD9 and Calnexin in the Exo. B, Uptake of BC cell-derived Exo by MCF-7/ADR cells (25 μm). Fluorescent phalloidin-FITC (green) was used to stain F-actin, and DAPI (blue) was used to stain the nuclei.

**
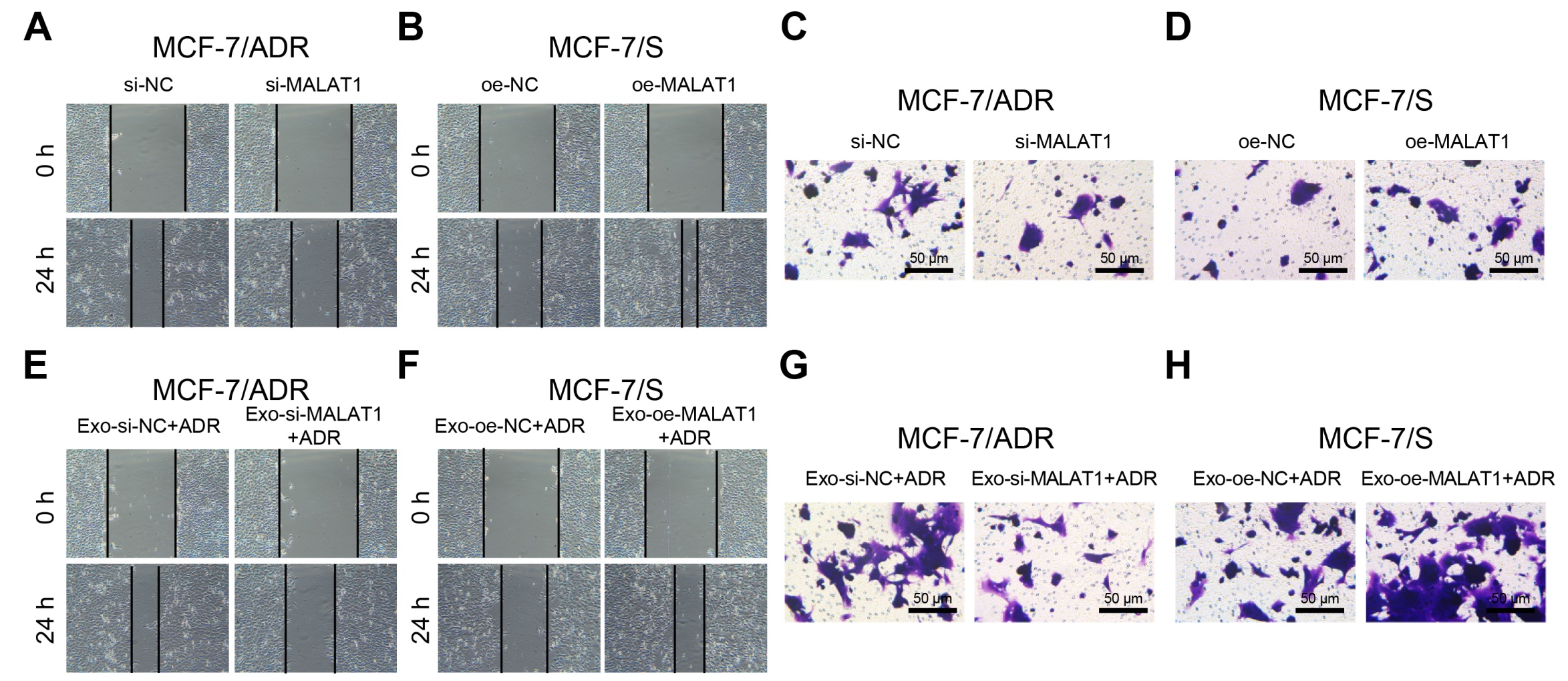
**

**Supplementary Figure 2** A, Scratch test of migration of MCF-7/ADR cells with MALAT1 silencing. B, Scratch test of migration of MCF-7/S cells with MALAT1 over-expression. C, Transwell assay detection of invasion of MCF-7/ADR cells with MALAT1 silencing (200 ×). D, Transwell assay detection of invasion of MCF-7/S cells with MALAT1 over-expression (200 ×). E, Scratch test of migration of MCF-7/ADR cells treated with Exo-si-MALAT1 + ADR. F, Scratch test of migration of MCF-7/S cells treated with Exo-oe-MALAT1 + ADR. G, Transwell assay detection of invasion of MCF-7/ADR cells treated with Exo-si-MALAT1 + ADR (200 ×). H, Transwell assay detection of invasion of MCF-7/S cells treated with Exo-oe-MALAT1 + ADR (200 ×).

**
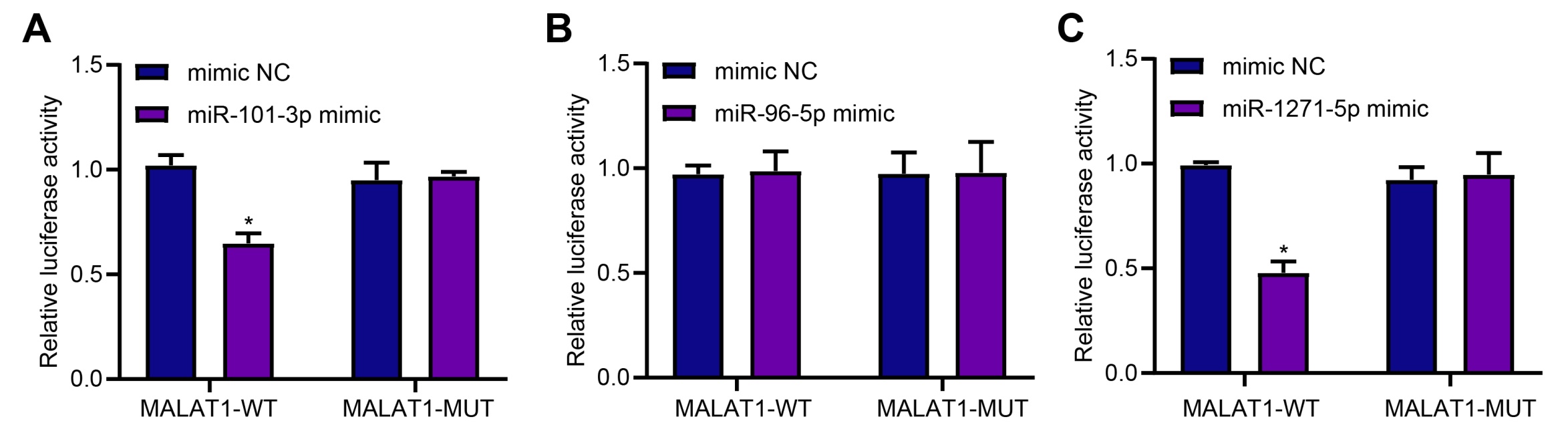
**

**Supplementary Figure 3** Dual-luciferase reporter gene assay on binding between MALAT1 and hsa-miR-101-3p (A), hsa-miR-96-5p (B), or hsa-miR-1271-5p (C).

**
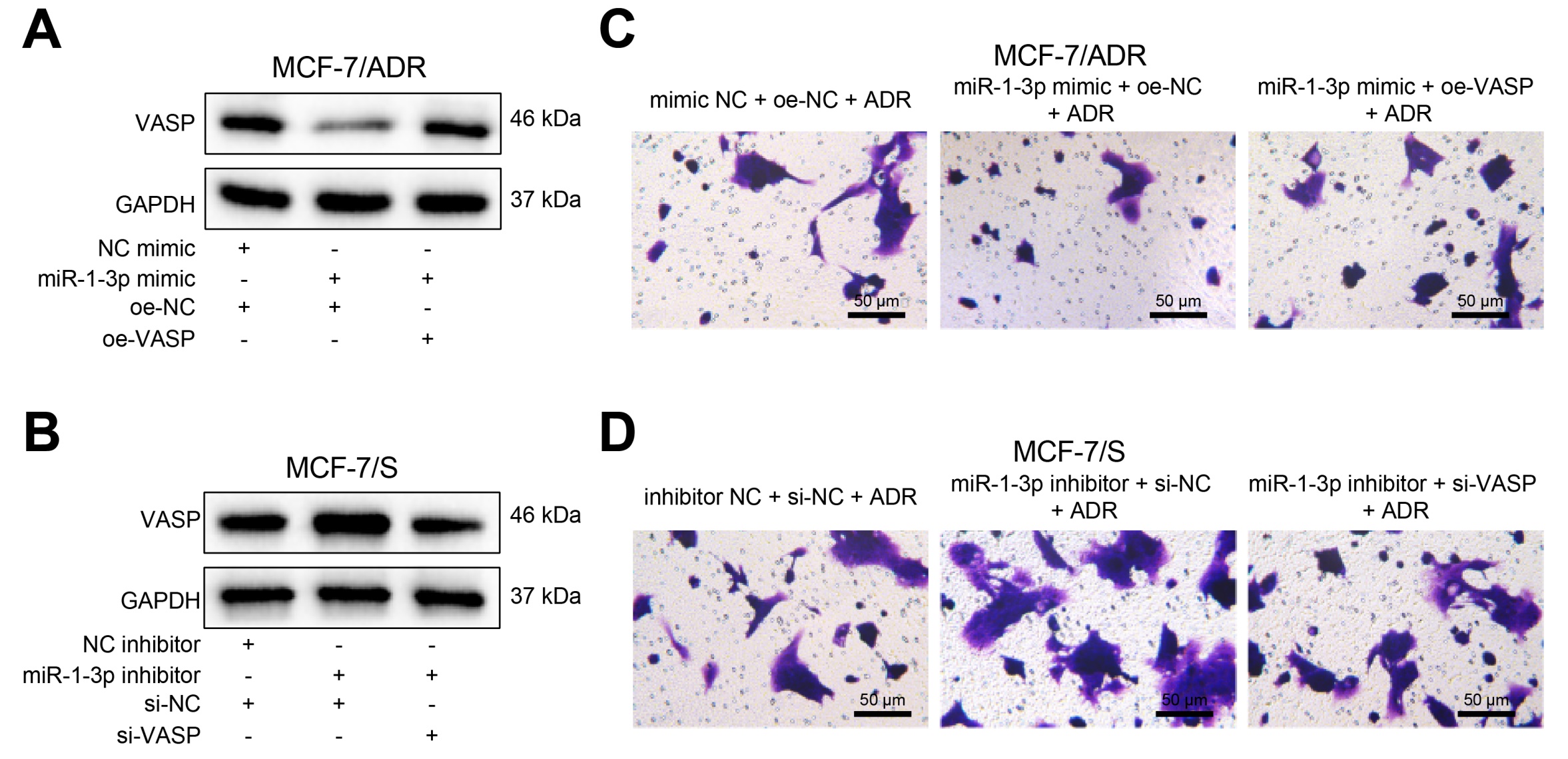
**

**Supplementary Figure 4** A, Western blot analysis of VASP protein band in MCF-7/ADR cells treated with miR-1-3p mimic or combined with oe-VASP. B, Western blot analysis of VASP protein in MCF-7/S cells treated with miR-1-3p inhibitor or combined with si-VASP. C, Transwell assay detection of invasion of MCF-7/ADR cells treated with miR-1-3p mimic or combined with oe-VASP (200 ×). D, Transwell assay detection of invasion of MCF-7/S cells treated with miR-1-3p inhibitor or combined with si-VASP (200 ×).
